# Succession of Bacteria and Archaea Within the Soil Micro‐Food Web Shifts Soil Respiration Dynamics

**DOI:** 10.1111/1462-2920.70007

**Published:** 2024-11-21

**Authors:** Mandip Tamang, Johannes Sikorski, Miriam van Bommel, Marc Piecha, Tim Urich, Liliane Ruess, Katharina Huber, Meina Neumann‐Schaal, Michael Pester

**Affiliations:** ^1^ Leibniz Institute DSMZ—German Collection of Microorganisms and Cell Cultures Braunschweig Germany; ^2^ Humboldt‐Universität zu Berlin, Institute of Biology, Ecology Group Berlin Germany; ^3^ University of Greifswald, Institute of Microbiology Greifswald Germany; ^4^ Braunschweig Integrated Centre of Systems Biology (BRICS) Braunschweig Germany; ^5^ Technical University of Braunschweig, Institute for Microbiology Braunschweig Germany

**Keywords:** grazing, microbial community, nematode, respiration, soil, trophic interactions

## Abstract

Bacterivorous nematodes are important grazers in the soil micro‐food web. Their trophic regulation shapes the composition and ecosystem services of the soil microbiome, but the underlying population dynamics of bacteria and archaea are poorly understood. We followed soil respiration and 221 dominant bacterial and archaeal 16S rRNA gene amplicon sequencing variants (ASVs) in response to top‐down control by a common bacterivorous soil nematode, *Acrobeloides buetschlii* , bottom‐up control by maize litter amendment and their combination over 32 days. Maize litter amendment significantly increased soil respiration, while *A. buetschlii* addition caused an earlier peak in soil respiration. Underlying bacterial and archaeal population dynamics separated into five major response types, differentiating in their temporal abundance maxima and minima. In‐depth analysis of these population dynamics identified a broad imprint of *A. buetschlii* grazing on dominant bacterial (*Acidobacteriota, Bacteroidota, Gemmatimonadota, Pseudomonadota*) and archaeal (*Nitrososphaerota*) ASVs. Combined bottom‐up control by maize litter and top‐down control by *A. buetschlii* grazing caused a succession of soil microbiota, driven by population changes first in the *Bacteroidota*, then in the *Pseudomonadota* and finally in the *Acidobacteriota* and *Nitrososphaerota*. Our results are an essential step forward in understanding trophic modulation of soil microbiota and its feedback on soil respiration.

## Graphical abstract

Nematode grazing and plant litter are important biotic control mechanisms of the soil microbiome. We show that major bacterial and archaeal taxa respond in five distinct ways to top‐down and bottom‐up controls, resulting in a succession of soil microbiota and shifts in soil respiration dynamics.

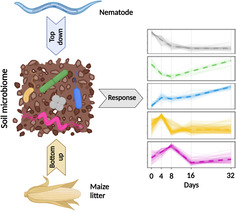

## 1 Introduction

The soil microbiome comprises the most diverse biological communities on Earth, encompassing at least 25% of our planet's biodiversity (Guerra et al. 2021) with tens of millions of bacterial, archaeal, fungal and microeukaryotic species (Orgiazzi et al. 2016). Interestingly, a recent meta‐analysis of 237 soils across six continents established that only 2% of bacterial phylotypes (about 500 in total numbers) found in soils consistently accounted for 41% of the soil bacterial communities worldwide (Delgado‐Baquerizo et al. 2018). Thus, relatively few bacterial taxa are dominating in numbers across soils globally. The eight most abundant bacterial phyla found in soils (in decreasing relative abundance) are typically represented by heterotrophic members of the *Pseudomonadota* (mainly *Alpha‐, Beta‐* and *Gammaproteobacteria*), *Actinobacteriota*, *Acidobacteriota*, *Planctomycetota*, *Chloroflexota*, *Verrucomicrobiota*, *Bacteroidota* and *Gemmatimonadota* (Bahram et al. 2018; Delgado‐Baquerizo et al. 2018). They are accompanied by the archaeal phylum *Nitrososphaerota* comprising typically 1%–5% of the total prokaryotic community (Leininger et al. 2006) and consisting of ammonia‐oxidising chemolithoautotrophs (Prosser and Nicol 2008; Alves et al. 2018).

It is evident that the ecology of the soil microbiome has direct impact on soil ecosystem services, for example, nutrient cycling, carbon sequestration and promoting plant growth to name a few (Brussaard 2012; Thakur and Geisen 2019). Also, a large share of global soil respiration is caused by the soil microbiome (35–69 Pg C/year) (Bond‐Lamberty, Wang, and Gower 2004; Tian et al. 2015) because of its inherent role in organic matter decomposition and mineralization (Tecon and Or 2017; Sokol et al. 2022). Therefore, the soil microbiome has a direct impact on carbon exchange between the land surface and the atmosphere (Bond‐Lamberty and Thomson 2010; Hashimoto et al. 2015). Four major mechanisms were identified to govern the ecology of the soil microbiome. As principal regulators, climatic conditions and abiotic soil properties such as pH and organic matter content were suggested (Fierer 2017). In addition, two biotic control mechanisms were identified, particularly at the finer spatial scale. Bottom‐up control is exerted by litter input and rhizodeposition, whereas top‐down control is driven by predators and viruses (Thakur and Geisen 2019; Sokol et al. 2022). While the importance of bottom‐up control, that is, plant input, is already well recognised and studied (Bulgarelli et al. 2012, 2013, 2015; Chaparro, Badri, and Vivanco 2013; Dawson et al. 2017; Brunel et al. 2020), the role of top‐down control by predation has received much less attention (Richter et al. 2019; Thakur and Geisen 2019; Sokol et al. 2022; Ruess 2024).

In particular, nematodes and protozoa are recognised as regulators of microbial community structure and to modulate nutrient mineralization of their microbial prey (Bonkowski, Villenave, and Griffiths 2009; Ferris 2010). Their grazing activity further accounts for the release of ammonium from microbial biomass, estimated as high as 32%–38% of the annual N‐mineralization in arable land (Whalen et al. 2013). Thus, eukaryotic grazers link the soil microbiome and its ecosystem functions with the faunal food web (Neher 2010; Yeates 2010; Heijboer et al. 2017) and hence the energy and matter flow to higher trophic levels. Nematodes constitute the most abundant (5 × 10^4^–27 × 10^6^ individuals m^−2^) and diverse (up to 200 species m^−2^) multi‐cellular organisms in soils (Yeates 2010; van den Hoogen et al. 2019; Potapov et al. 2022), with established functional groups at each trophic level, feeding on bacteria, fungi or roots as well as microfauna (Yeates et al. 1993). Bacterivorous nematodes represent the functionally dominating group within soil nematode communities, typically accounting for > 40% of all nematode counts across different terrestrial biomes worldwide (van den Hoogen et al. 2019).

Nematodes feeding on bacteria are often described as generalists that randomly ingest any bacteria by their filter‐feeding habit (Thakur and Geisen 2019). However, there is increasing evidence that they do differentiate their prey bacteria based on morphology, cell‐wall characteristics, mucus production or metabolite concentration (Bjørnlund et al. 2006; Liu et al. 2017). Several studies reported preference for Gram‐negative over Gram‐positive bacteria, likely due to the thinner cell wall and easier digestibility (Salinas et al. 2007; Yu et al. 2015; Liu et al. 2017). Also, a high water content, a high C/N ratio as a proxy for food quality as well as the respiration rate as a proxy for metabolic activity were identified as positive prey selection criteria of bacterivorous nematodes (Liu et al. 2017; Richter et al. 2019; Hemmerling, Ackermann, and Ruess 2023). Differing life‐history traits among nematode species are yet another differentiating factor and are commonly expressed as the coloniser‐persister (cp) value (Bongers 1990, 1999). The cp‐value is categorised into five classes, cp1—cp5. Colonisers that are characterised by high reproduction rates receive a low cp value, while persisters that reproduce slowly are allocated to high cp‐values (Bongers 1990, 1999). For example, enrichment opportunists such as the model nematode *Caenorhabditis elegans* (cp 1) are only active under food‐rich conditions (Bongers 1990, 1999) as supported by its wide tubular stoma and consumption of food particles through continuous pumping of the oesophagus (pharynx) (Hemmerling, Ackermann, and Ruess 2023). In contrast, general opportunists that are common in many soils, such as the nematode *Acrobeloides buetschlii* (cp 2), can live under food‐poor conditions (Bongers 1990, 1999). In the latter, this goes along with a narrow, muscular stoma and a lower pumping frequency with its pharynx (Hemmerling, Ackermann, and Ruess 2023).

Grazing activities by bacterivorous nematodes alter soil respiration (Xiao et al. 2014; Zhu et al. 2018; Richter et al. 2019) and positively effect organic N mineralization (Anderson et al. 1983; Bouwman et al. 1994; Holajjer, Kamra, and Singh 2016). In combination with their feeding preferences, this translates directly into changes on the total abundance and community composition of soil bacteria (Xiao et al. 2014; Jiang et al. 2017; Brondani et al. 2022). However, the underlying population dynamics of individual bacterial and archaeal species are only rudimentarily explored and are typically only described in terms of beta‐diversity changes focusing on end‐points of the respective experiments. Here, we followed time‐resolved population dynamics of bacterial and archaeal 16S rRNA gene amplicon sequencing variants (ASVs) at the absolute abundance level in response to top‐down control by a common bacterivorous soil nematode, *A. buetschlii* , bottom‐up control by maize litter addition and the combination of both. We hypothesize that (i) different combinations of grazing pressure and substrate availability differentially modulate microbial community composition dynamics, (ii) the underlying population changes of individual bacterial and archaeal ASVs can be grouped into major response types and (iii) identified time‐resolved response types can be conceptualised into a succession model.

## 2 Experimental Procedures

### 2.1 Soil

The Dikopshof agricultural site (50°48′21″ N, 6°59′9″ E) is a long‐term field experiment that is maintained by the University of Bonn, Germany. It is located at 62 m above sea level with an average annual temperature and precipitation of 10.5°C and 688 mm, respectively. The dominant soil type is Haplic Luvisol composed of 68.9% silt, 15.9% sand and 15.1% clay. The soil has a pH (0.01 M CaCl_2_) of 6.3, a total organic carbon content of 0.74% and a total nitrogen content of 0.08%. The site is managed as a 5‐year crop rotation of sugar beet ( *Beta vulgaris* L.), winter wheat ( *Triticum aestivum* L.), winter rye ( *Secale cereale* L.), Persian clover ( *Trifolium resupinatum* L.) and potatoes ( *Solanum tuberosum* L.). On the plots sampled, farmyard manure has been applied annually as sole fertiliser at a rate of 20 tons per ha and crop (Abdalla et al. 2022). The nematode community of the used soil was dominated by bacterivorous nematodes (79%) followed by plant parasites (12%), fungivorous nematodes (8%), omnivorous nematodes (1%) and predatory nematodes (1%) (van Bommel et al. 2024). Farmyard manure‐fertilised soil was collected in August 2021. Soil was taken randomly from the upper 20 cm. After transport to the laboratory, soil samples were air dried at room temperature (RT) for at least 14 days, homogenously mixed, sieved (2 mm mesh) and stored at RT. This served to reduce the autochthonous nematode community.

### 2.2 Nematodes

Nematode populations during microcosm incubations and at the natural site were determined using a modified wet funnel method (Ruess 1995). In brief: Nematodes were extracted from 20 to 22 g soil at 20°C for 24 h, followed by a step‐wise heat extraction (+5°C h^−1^, up to 45°C). Animals were then fixed in 4% formaldehyde and stored at 4°C. Total nematode counts were determined at 40‐fold magnification with a Zeiss Axioscope 5 microscope. Subsequently, 10% of the extracted nematodes were used to determine the composition of trophic groups and specifically the genus *Acrobeloides* at 400‐fold magnification. Trophic groups were assigned according to Yeates et al. (1993).

Monoxenic stock cultures of the bacterivorous nematode *A. buetschlii* were maintained at 15°C on potato dextrose agar (Carl Roth, Karlsruhe, Germany) inoculated with the ascomycete *Chaetomium globosum*. The agar and the compounds secreted by *C. globosum* served as food for *A. buetschlii* . For grazing experiments, *A. buetschlii* individuals were extracted from plates at 20°C for 24 h using a modified wet funnel method after Baermann (1917). Extracted nematodes were surface sterilised with 1 mL 0.01% HgCl_2_‐solution (w/v) for 3 min, followed by washing with 3 mL autoclaved mineral water (Volvic, Danone waters GmbH, Frankfurt, Germany) for three times. Thereafter, nematodes were stored overnight in autoclaved mineral water at 4°C before onset of experiments.

### 2.3 Soil Microcosm Setup

Prior to experiments, stored dry soil was pre‐incubated for 10 days with 14% (w/v) demineralized water at RT to reactivate the soil microbiome. Thereafter, soil microcosms were set up as described previously (Richter et al. 2019). Per microcosm, 50 g dry weight (DW) of pre‐incubated soil was placed in a 100 mL crimp neck flask (Rotilabo, Carl Roth) and sealed air tight with crimp caps (Rotilabo, Carl Roth). Each microcosm was provided continuously with ambient air at a rate of approximately 275 mL min^−1^ using a vacuum pump (Sera air 550 R plus, Heinsberg, Germany). The inflowing air was first humidified by passing through distilled water and filter‐sterilised by a 0.2 μM filter (Sartorius minisart, Göttingen, Germany) before entering the microcosm. The outflowing air was passed through a separate vial containing 15 mL of 1 M KOH to trap CO_2_ produced in the microcosms. Microcosms were maintained in a growth chamber (PERCIVAL E−41L2, Percival Scientific, Perry, IA, USA) at 3 ppm CO_2_, 33% relative humidity and 20°C in the dark. Microcosms of different treatment combinations were randomly connected to air supply units and randomly distributed in the growth chamber. The water content within microcosms was kept at 13% without *A. buetschlii* addition and at 16% with *A. buetschlii* addition (first 4 days at 20%) by regularly injecting autoclaved mineral water with a disposable sterile syringe (Omnifix 10 mL, Braun, Melsungen, Germany). The slightly higher water content in incubations with *A. buetschlii* resulted from initial nematode addition.

Treatments of microcosms followed a 2‐factorial design: with or without maize litter and with or without *A. buetschlii* , resulting in four treatment combinations (Figure S1). Maize litter (IsoLife, Wageningen, The Netherlands) originated from cob, leaf and stem (milled < 2 mm) and was supplied as 0.76 mg (g soil DW)^−1^, which was roughly equivalent to fourfold of microbial biomass in the used soil. Nematodes were extracted from monoxenic stock cultures as described above and added at a final amount of 435 ± 35 *A. buetschlii* individuals per microcosm. This corresponded to the number of bacterivorous nematodes [8 ± 4 individuals (g soil DW)^−1^, mean ± standard deviation] detected beforehand in the used soil (van Bommel et al. 2024). Each treatment was set up in five replicates per timepoint that were sampled destructively at days 4, 8, 16 and 32. Samples at day 0 were shared in the treatments with and without *A. buetschlii* addition.

### 2.4 Soil Respiration

Soil respiration was determined as described previously (Anderson and Domsch 1975; Richter et al. 2019). In brief: CO_2_ trapped in 1 M KOH‐containing vials was determined every 2–4 days. Background CO_2_ was determined by using empty microcosms without soil (blanks). Strontium carbonate (SrCO_3_) was precipitated out of 3 mL CO_2_‐containing KOH solution using 3 mL 0.5 M SrCl_2_. The precipitate containing solution was amended with 2 drops of 1% phenolphthalein solution and titrated with 0.1 M HCl until colour change. The trapped CO_2_ was calculated according to the following equation:

$$CO_{2}\left[\frac{\mathrm{\mu g}}{g\;\text{soil}\;\mathrm{dry}\;\text{weight}\cdot h}\right]=\frac{\left({\mathrm{HCl}}_{\text{blank}}-{\mathrm{HCl}}_{\text{sample}}\right)\cdot 2.2\cdot 1000}{\text{soil}\;\mathrm{dry}\;\text{weight}\cdot \text{time}}$$

where HCl_blank/sample_ are the volumes of HCl consumed by blanks and samples (mL) and 2.2 is the conversion factor of 0.1 M HCl to 2.2 mg CO_2_. The factor 1000 was used to convert mg into μg. Generalised additive modelling (GAM) of soil respiration rates was done in R package mgcv v. 1.8‐41 (Wood 2017). GAM model development was performed as recommended previously (Pedersen et al. 2019). Model assumptions were verified as suggested previously (Zuur and Ieno 2016) by plotting residuals versus fitted values via gratia::appraise() v. 0.8.1.38 (Simpson 2024; Figure S2).

### 2.5 Nucleic Acid Extraction

Upon destructive sampling of microcosms, soil samples were frozen immediately in liquid nitrogen (N_2_) and stored at −80°C. Three out of five soil sample replicates per timepoint and maize/*A. beutschlii* treatment combination were randomly selected and used in downstream analyses, resulting in a total of 54 samples. DNA was extracted from approx. 2 g of soil using the RNeasy PowerSoil Total RNA kit in combination with the RNeasy PowerSoil DNA elution kit (Qiagen, Hilden, The Netherlands). DNA concentrations were determined fluorometrically using a Qubit 4 (Thermo Fisher Scientific, Waltham, MA, USA).

### 2.6 Quantitative PCR

Quantification of total bacterial and archaeal 16S rRNA genes was done as described previously (Dawson et al. 2017) using a quantitative PCR (qPCR) assay. The assay was based on the universal primer pair 1389F (5′‐TGYACACACCGCCCGT‐3′) and 1492R (5′‐GGYTACCTTGTTACGACTT‐3′) and conducted in 20 μL reactions on a qTOWER^3^G (Analytik‐Jena, Jena, Germany). PCR reactions consisted of 10 μL of LightCycler 480 SYBR Green I master mix (Roche, Penzberg, Germany), 0.4 μL of each forward and reverse primers (10 μM), 5 μL of template DNA (1.6–6.5 ng) and 4.2 μL of PCR‐grade DNA‐free water (Molzym GmbH and Co. KG, Bremen, Germany). After testing a dilution series of template DNA, 1:50 dilutions were used to avoid partial PCR inhibition by co‐extracted compounds. An amplified 16S rRNA gene fragment of *Escherichia coli* strain K12 cloned into the pCR 4‐TOPO vector (Invitrogen, Waltham, MA, USA) was used as standard curve in the range 10^2^–10^7^ copies μL^−1^ (R^2^ = 0.99). Amplification was carried out using an initial denaturation at 95°C for 10 min followed by 46 cycles of denaturation at 95°C for 30 s, annealing at 52°C for 30 s and elongation at 72°C for 30 s. PCR efficiencies were on average 90.0 ± 2.8. Specificity of qPCRs was checked by running a melting curve after each qPCR run.

### 2.7 16S rRNA Gene Amplicon Sequencing and Sequence Processing

Total bacterial and archaeal 16S rRNA genes were amplified using a procedure described before (Herber et al. 2020) with minor modifications. In brief: 5 ng of extracted DNA were used as template to amplify the V4 region of bacterial and archaeal 16S rRNA genes using universal primers 515F (5′‐GTGYCAGCMGCCGCGGTAA‐3′) and 806R (5′‐GGACTACNVGGGTWTCTAAT‐3′) (modified from Caporaso et al. 2011) and the Platinum Hot Start PCR master mix (Invitrogen). Polymerase chain reaction (PCR) was conducted under the conditions of an initial denaturation of 94°C for 3 min followed by 10 cycles of denaturation at 94°C for 45 s, annealing at 50°C for 60 s and elongation at 72°C for 90 s. Subsequently, PCR products were purified using Agencourt AMPure XP magnetic beads (Beckman Coulter, Indianapolis, IN, USA), eluted in 24 μL PCR‐grade water and 20 μL used as template for a second PCR with 14 cycles for the introduction of barcodes and Illumina sequencing adaptors. PCR conditions were the same as in the first PCR. After pooling equimolar amounts of template DNA, amplicon sequencing was performed at the Leibniz Institute DSMZ—German Collection of Microorganisms and Cell Cultures, Germany on the Illumina MiSeq platform using paired‐end sequencing (2 × 300 bp, MiSeq reagents kit v3). Amplicon reads were processed using Qiime2 version 2023.5 (Bolyen et al. 2019), including quality control, ASV clustering and de novo chimera filtering using DADA2 (Callahan et al. 2016). Processed samples had on average 114,468 paired‐end reads, with 90% of all samples having between 57,669 and 166,633 paired‐end reads (5% and 95% quantiles, respectively). ASVs were taxonomically classified using the naïve Bayes classifier in Qiime2 and the Silva database v.138.1 (Quast et al. 2013).

### 2.8 Alpha and Beta Diversity of Bacterial and Archaeal Amplicon Sequencing Variants

Quality‐controlled ASV and qPCR data analyses were performed in R version 4.2.2 (R‐Core‐Team 2022). Graphs were generated using R package ggplot2 v.3.4.4 (Villanueva and Chen 2019). Sampling coverage (Chao and Jost 2012) was estimated based on the Qiime2‐processed ASV table in R package iNEXT v. 3.0.0. (Hsieh, Ma, and Chao 2016). Coverage equalled for all samples 1 indicating close to complete sampling coverage. As a consequence, no downstream normalisation based on rarefaction was applied. If indicated, relative abundances were transformed to absolute abundances by multiplying with the respective total 16S rRNA gene copy numbers as obtained by qPCR. Alpha diversity indices based on Hill number analysis were obtained on absolute abundance transformed ASV tables using the R package hilldiv v.1.5.1 (Alberdi and Gilbert 2019a). The alpha‐gambin value as a parameter describing ASV abundance distributions (McGill et al. 2007) was obtained on read count ASV tables using the R package gambin v. 2.5.0 (Matthews et al. 2014). A higher alpha‐gambin value reflects a flatter ASV abundance distribution, indicating a more even distribution of ASV abundances. To test for significant alpha diversity changes an analysis of variance (ANOVA) was performed using the R package stats v. 4.2.2. Significant ANOVA results were subjected to post hoc comparisons of group means using the R packages multcomp v.1.4‐25 (Hothorn, Bretz, and Westfall 2008) and sandwich v.3.0‐2 (Zeileis 2006; Zeileis, Köll, and Graham 2020) as described previously (Herberich, Sikorski, and Hothorn 2010). Beta diversity analysis followed the procedure recommended by Gloor et al. (2017). Considering the compositional nature of amplicon data (Gloor et al. 2017), the ASV table with zero‐imputed read count values was subjected to a centred log‐ratio transformation (CLR). Zeros were imputed in the read count ASV table using the geometric Bayesian multiplicative (GBM) method of the cmultRepl function implemented in the R zCompositions package v. 1.5.0‐4 (Palarea‐Albaladejo and Martín‐Fernández 2015). Thereafter, an Aitchison distance matrix was obtained and used for both a principle component analysis (PCA) ordination and for variance partitioning using the R package vegan v. 2.6‐6.1 (Oksanen et al. 2024).

### 2.9 Classification of ASV Abundance Changes Into Response Types

Classification of time‐resolved ASV abundance changes into response types was based on numerically dominant ASVs as defined by Hill number ^2^ *D* (Alberdi and Gilbert 2019b). Briefly: Hill number ^2^ *D* is an established measure in ecology on the effective number of dominant taxa in a sample and is derived from the inverse of the Simpson index (Alberdi and Gilbert 2019b). Its big advantage is that it considers both, ASV richness and evenness. As both may differ between different samples, the Hill number concept should not be confused with the simpler cutoff of 0.1% relative abundance previously introduced to microbial ecology to differentiate low‐abundance taxa (rare biosphere) from so called ‘abundant’ taxa (Sogin et al. 2006; Pedrós‐Alió 2012; Lynch and Neufeld 2015). In the Hill number concept, ASV richness (^0^ *D*), abundant ASVs (^1^ *D*) and dominant ASVs (^2^ *D*) are differentiated and their respective cutoff is depending on the ASV richness and evenness in a given sample.

For response type analysis, ASVs had to be dominant in at least one of the 54 samples used for molecular analyses. In addition, dominant ASVs had to be detected in at least two out of three replicates per timepoint across all four experimental treatment combinations. This resulted in a subset of 359 dominant ASVs, which had a relative abundance of 0.0035 to 7.9% (mean = 0.19%; median = 0.09%) across all samples. To discern responsive ASVs among this subset, the latter were subsampled based on their absolute abundance range, that is, minimum to maximum at any given timepoint. Following the Pareto principle, only ASVs were considered for downstream analysis that represented 80% of the total absolute summed abundance ranges across all treatments. This resulted in 221 dominant responsive ASVs, which were further grouped into response types. To foster comparability across all responsive ASVs, absolute abundances of each ASV were first scaled to a mean = 0 and a standard deviation = 1 using the R package stats v. 4.2.2. Thereafter, scaled response patterns were subjected to k‐means clustering. An initial pre‐screening analysis was performed using three different clustering methods as implemented in the R package factoextra v. 1.0.7 (Kassambra and Mundt 2020). The method ‘gap_stat’ was used with the additional arguments nstart = 25 and nboot = 50. Both, the ‘wss’ (within cluster sums of squares) and the ‘silhouette’ method suggested four optimal clusters, whereas the ‘gap_stat’ (gap statistic) suggested nine optimal clusters. As a result, the analysis of response types was initiated with a k‐means clustering using nine clusters. Visual inspection identified highly similar response types, which were further grouped into a final set of five different response types, which was justified by the suggested 4–9 optimal clusters.

## 3 Results

### 3.1 Addition of the Bacterivorous Nematode *A. buetschlii* Speeds Up Soil Respiration

Soil microcosms were set up to examine biotic controls of the bacterial and archaeal community in a typical agricultural soil. The nematode community in this soil is strongly dominated by the trophic group of bacterivorous nematodes (ca. 79%). Top‐down control by grazing was investigated by addition of the bacterivorous nematode *A. buetschlii* . Bottom‐up control by plant input was investigated by addition of maize litter (Figure). First, the autochthonous nematode community was strongly reduced from 11 ± 5 to 0.3 ± 0.0 individuals (g soil DW)^−1^ by storing soil in a dried state and rewetting for 10 days before the onset of the experiment. This was necessary to discern the effect of *A. buetschlii* addition. Rewetting served to activate the soil microbiome before the onset of the experiment and to avoid a Birch effect in soil respiration during the actual incubations.

**FIGURE 1:**
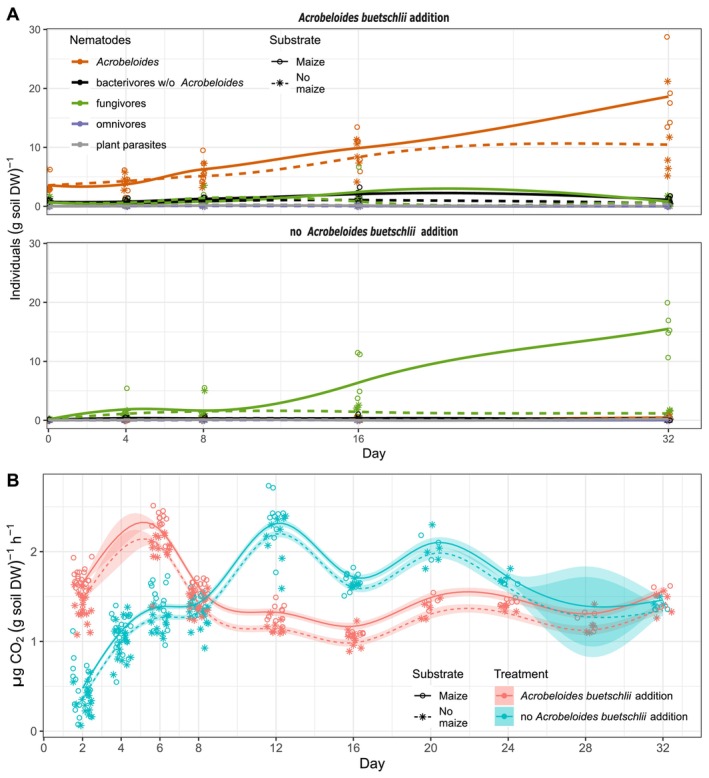
(A) Population dynamics of nematode trophic groups and specifically the genus *Acrobeloides* in response to addition of the bacterivorous nematode *A. buetschlii* and maize litter. Individual nematode counts are given as symbols under the given treatment. (B) Soil respiration rate in response to addition of the bacterivorous nematode *A. buetschlii* and maize litter. Individual measurements are given as symbols under the given treatments. In addition, a generalised additive model (GAM) was fitted to the measured values and shown as continues solid or dashed lines. Details of the GAM are given in Tables S1 and S2 as well as in Figure S2. The standard error is shown as shades next to the GAM model line.

Throughout the experiment, population dynamics of *Acrobeloides* nematodes in specific and of nematode trophic groups in general were followed. *Acrobeloides* nematodes dominated the nematode community composition in *A. buetschlii* addition setups irrespective of maize addition. After addition at day 0, they could be recovered at an average population density of 3.6 ± 1.5 ind. (g soil DW)^−1^, which stayed stable within the first 4 days of incubations—with and without maize litter addition. Thereafter, *Acrobeloides* populations started to increase, which was more pronounced with maize litter than without. At day 32, they reached final population densities of 18.6 ± 6.1 and 10.5 ± 6.5 ind. (g soil DW)^−1^ with and without maize litter addition, respectively (Figure 1A). Other bacterivorous nematodes and nematodes of other trophic groups did not establish substantial populations in these soil microcosms (Figure 1A).

In microcosms without *A. buetschlii* addition, total nematode densities were much lower at day 0 with an average of 0.26 ± 0.04 ind. (g soil DW)^−1^. The share of *Acrobeloides* populations was 0.01 ± 0.00 ind. (g soil DW)^−1^. In contrast to microcosms with *A. buetschlii* addition, fungivorous nematodes started to establish as dominating trophic nematode group in these microcosms throughout the duration of the experiment (Figure 1A). In treatments without maize addition, they established only very small populations with an average of 1.1 ± 0.7 ind. (g soil DW)^−1^. In treatments with maize, fungivorous nematode populations were also low throughout the first 8 days of incubation with an average of 1.8 ± 2.0 ind. (g soil DW)^−1^. Thereafter, they started to increase and reached final densities of 15.5 ± 3.4 ind. (g soil DW)^−1^. Other trophic groups including bacterivorous nematodes did not establish substantial populations in these soil microcosms. The share of *Acrobeloides* populations was on average 0.2 ± 0.2 ind. (g soil DW)^−1^ throughout the 32 days of incubation, irrespective if maize litter was added or not (Figure 1A).

Soil microcosms with added *A. buetschlii* showed a clear shift of the maximum rate of soil respiration to an earlier time point (from day 12 to day 6, Figure 1B) and a decreased oscillation of CO_2_ release from soil. Presence or absence of added *A. buetschlii* nematodes also explained best dynamics of soil respiration over the examined 32 days (Table S1). In contrast, addition of maize litter significantly (*p* < 0.05) increased soil respiration rates, whereas the addition of *A. buetschlii* nematodes or the combined effect of both had no such effect (Table S2).

### 3.2 Grazing and Substrate Availability Alter Bacterial and Archaeal Community Composition

Total bacterial and archaeal abundances, ASV richness and the number of dominant ASV as defined by Hill number ^2^ *D* (Alberdi and Gilbert 2019b) were on average 2.35 ± 0.53 × 10^8^ 16S rRNA gene copies per gram dry soil, 2.06 ± 0.39 × 10^3^ ASVs, and 1.54 ± 0.38 × 10^2^ ASVs at the onset of the experiment, respectively (Figure S3). Total bacterial and archaeal abundances changed only significantly over time when both bacterivorous *A. buetschlii* and maize litter were added (Tables S3 and S4). ASV richness, the number of dominant ASVs and the rank abundance distribution of the bacterial and archaeal community did not change significantly throughout the incubation in any of the incubation types (Tables S3 and S4).

**FIGURE 2:**
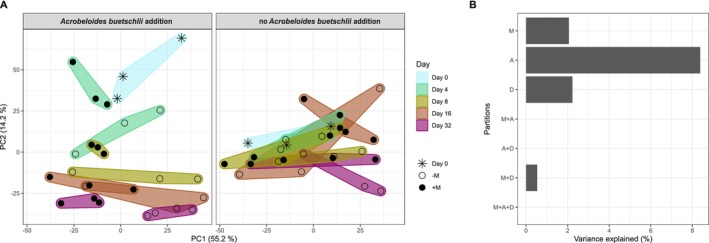
(A) Time‐resolved beta diversity changes of the bacterial and archaeal community in response to addition of the bacterivorous nematode *A. buetschlii* (indicated in the panels) and maize litter (+M: added maize litter; −M: no addition of maize litter). The analysis was based on a principal component analysis (PCA) of the Aitchison distance metric. (B) Corresponding variance partitioning of beta diversity changes of the bacterial and archaeal community. M: Maize litter addition, A: *A. buetschlii* addition, D: Incubation time. Interactions of individual explanatory variables are indicated with the plus sign.

While alpha diversity metrics stayed rather stable throughout the experiment, there was an effect of both bacterivorous *A. buetschlii* and maize litter addition on the overall community composition of bacteria and archaea (Figure 2A). Most of these community changes occurred within the first 16 days of the experiment and were more pronounced in incubations with *A. buetschlii* addition (Figure 2A, Figure S4). There were little changes in the remaining 16 days irrespective of treatment (Figure S4). Variance partitioning of observed beta diversity changes could explain 13.2% of the variance in the data. *A. buetschlii* addition had the largest effect, followed by incubation time and maize litter addition. Interactions among these parameters had a negligible effect on observed beta diversity changes (Figure 2B).

### 3.3 Five Ways Bacteria and Archaea Numerically Respond to Top Down and Bottom Up Control

Abundance changes of individual ASVs in response to *A. buetschlii* addition, maize litter addition and the combination of both were analysed by grouping ASVs into time‐resolved response types. The focus was put on dominant ASVs as defined by Hill number ^2^ *D* as these can be considered to drive the major ecosystem functions. This approach was preferred over classical differential abundance testing since it considered (i) temporal dynamics across multiple time points (ii) focused on populations with major absolute (and not relative) abundance changes and (iii) did not suffer from *p*‐value inflation due to massive parallel, pairwise testing and the resulting sensitivity loss of detecting biologically meaningful population changes.

**FIGURE 3:**
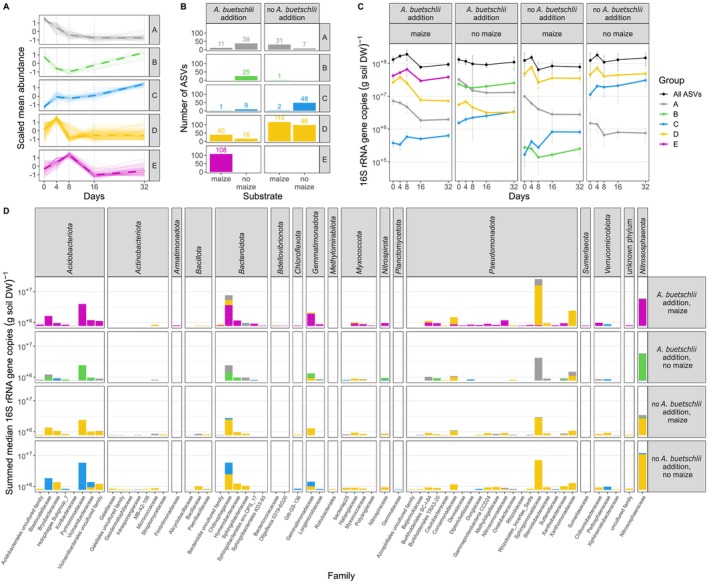
Classification of ASV abundance changes into response types across the different treatment combinations of the bacterivorous nematode *A. buetschlii* (top‐down control) and maize litter addition (bottom‐up control). (A) Response types as identified by k‐means clustering of absolute abundances scaled to a mean = 0 and a standard deviation = 1. For each response type, thin solid lines indicate scaled abundance changes of representing ASVs and the dashed thick line indicate the respective median. (B) Number of dominant responsive ASVs belonging to the individual response types under the different treatment combinations. (C) Contribution of response types to overall absolute abundance changes of the total bacterial and archaeal community. The mean ± standard deviation is provided for 2–3 replicates. (D) Summed median abundances of ASVs across all incubation timepoints. ASVs were grouped at the family level and according to the different response types under the different treatment combinations. For family names not validly published according to the Prokaryotic Code of Nomenclature, the next higher validly published rank name has been added. In the absence of an addition, the next validly published rank is the phylum level (except for ASVs that could not be affiliated to any known phylum).

Five different response types at the population level were identified, which were labelled from A to E (Figure 3A). Under bacterivorous *A. buetschlii* (A) and under maize (M) litter addition (+A/+M), response type E was dominating in terms of number of ASVs and contribution to overall abundance changes of total bacteria and archaea (Figure 3B,C). It was characterised by an intermediate abundance peak at day 8 and decline to starting point values towards day 32. In the parallel treatment without *A. buetschlii* but maize litter addition (−A/+M), response type D became dominant in terms of ASV numbers and contribution to overall abundance changes. Interestingly, response type D was very similar to response type E but the intermediate abundance peak was shifted forward to day 4 (Figure 3B,C). In the treatment with *A. buetschlii* but without maize litter addition (+A/−M) yet two other response types, type A and B, were dominating. Response type A was characterised by a steady decline in abundance over time, while response type B was characterised by an intermediate abundance minimum at day 8 and a recovery to starting point values at day 32 (Figure 3B,C). In the control treatment without *A. buetschlii* and maize litter addition (−A/−M), response type D was dominating again, as was the case in the treatment without *A. buetschlii* but with maize litter addition (−A/+M). In addition, response type C, which is characterised by a steady abundance increase over time, became dominant towards the end of the incubation as well (Figure 3B,C).

### 3.4 Soil Bacteria and Archaea Switch Between Response Type Depending on Treatment

**FIGURE 4:**
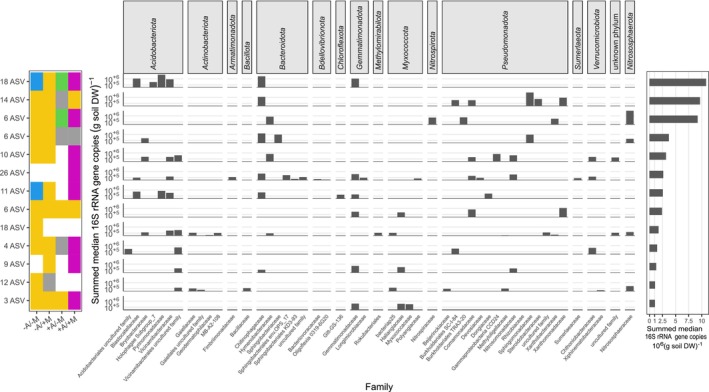
Response type changes of dominant response ASVs across all four treatments. The colour code corresponds to the response type shown in Figure 3. If left blank, ASVs did not group into any of the five response types under the given treatment but rather were temporally stable in abundance changes. The number of ASVs belonging to a certain response type pattern is indicated to the left. The taxonomic affiliation of respective ASVs and their median absolute abundance across all treatments and time points is shown in the centre. The median total abundance correlated highly significantly with the mean total abundance (Pearson's *R* ^2^ = 0.997, *p*‐value < 2.2 × 10^−16^). The median total abundance was chosen to indicate the base line of the total abundance of the respective ASV throughout the entire experiment. The summed median abundance of all ASVs belonging to the same response type pattern is shown to the right. Only response type patterns integrating over the majority of dominant response ASVs, that is, 80.9% of all summed median abundances, are shown. Summed median abundances of all response type patterns are shown in Figure S5 and the respective ASVs are given in Table S5. M: Maize litter, A: *A. buetschlii* , + and − signs indicate presence and absence, respectively.

The +A/+M treatment was clearly dominated by response type E (abundance maximum at day 8). This response type was spanning ASVs across 13 phyla with representatives of the *Acidobacteriota* (families *Blastocatellaceae* and *Pyrimonadaceae*), *Bacteroidota* (*Chitinophagaceae*), *Gemmatimonadota* (*Gemmatimonadaceae*) and *Nitrosphaerota* (*Nitrososphaeraceae*) dominating (Figure 3D). Except for the archaeal *Nitrososphaerota* (see below), most other dominant ASVs represented bacterial groups with a typically heterotrophic lifestyle. Interestingly, ASVs representing response type E under the +A/+M treatment switched to different response types under other treatment combinations. This resulted in treatment‐dependent response type patterns. For example, *Acidobacteriota* ASVs with large average abundances switched to an earlier abundance peak at day 4 in the −A/+M treatment (response type D), to an abundance decline towards day 8 and subsequent recovery (response type B) in the +A/−M treatment, and to a steady abundance increase (response type C) in the −A/−M treatment (Figure 4, Table S5). The second most important response type in the +A/+M treatment was response type D. In contrast to response type E, this response type was clearly dominated by *Pseudomonadota* ASVs, in particular representatives of the *Sphingomonadaceae*, *Xanthomonadaceae* and *Comamonadaceae* (Figure 3D). Here, the response type stayed quite stable throughout the different treatment types with the only exception being the +A/−M treatment, where a constant decline (response type A) was observed for some of these dominant ASVs (Figure 4, Table S5). In total, 56 of such response type combinations or patterns could be observed in our data set (Figure S5) with 13 of those representing > 80% of the summed median abundances of all dominant ASVs that were grouped into response types (Figure 4). The four most prominent response type patterns in terms of summed abundance had in common that their representing ASVs showed a decrease in abundance in the first 8 days in the +A/−M treatment (response type A and B) but showed the opposite behaviour with an abundance peak at day 4 in the −A/+M treatment (response type D) (Figure 4). They mainly differentiated in their response to the +A/+M treatment and to a minor extent in their behaviour in the −A/−M control treatment (Figure 4).

Responsive microorganisms with a specialised lifestyle were bacteria and archaea belonging to the functional guild of nitrifiers, that is, chemolithoautotrophic microorganisms converting ammonia via nitrite to nitrate. Especially *Nitrososphaerota* ASVs, which all belonged to typical soil ammonia oxidising archaea within the *Nitrososphaeraceae* (Pester et al. 2012; Alves et al. 2018; Ngugi et al. 2023) constituted one of the most abundant and responsive groups. While largely belonging to the dominant response type E in the +A/+M treatment, they clearly switched to response type B when maize litter was lacking. If *A. buetschlii* was not added, they switched to response type D irrespective of maize litter addition (Figure 3D, Figure 4). The same response pattern dynamics were observed for *Nitrospirota* ASVs affiliated to the genus *Nitrospira* that comprises typical soil nitrite‐oxidising bacteria and complete ammonia oxidizers (Daims, Lücker, and Wagner 2016; Figure 3D). Ammonia oxidising bacteria affiliated to the *Nitrosomonadaceae* (*Pseudomonadota*) constituted the third group within this functional guild, yet at smaller population size as compared to the *Nitrososphaeraceae* and *Nitrospira* ASVs. As the other nitrifiers, they belonged to response type E in the +A/+M treatment and most shifted to response type D if *A. buetschlii* was not added (−A/+M). In contrast to the other nitrifiers, the majority of *Nitrosomonadaceae* ASVs did not show relevant abundance changes in the +A/−M and −A/−M treatments (Figure 4, Table S5).

## 4 Discussion

### 4.1 The Broad Food Range of a Bacterivorous Nematode Spans Two Domains of Life

Adult nematodes of *A. buetschlii* and other bacterivorous species can consume about 10^5^–10^6^ bacterial cells daily (Venette and Ferris 1998). Their prey is essentially sucked in by the pumping action of the pharynx and hence their feeding behaviour can be regarded as filter‐feeding (Avery and Shtonda 2003; Rønn, Vestergård, and Ekelund 2012). However, binary interaction assays established that bacterivorous nematodes can display food preferences among prey bacteria (Salinas et al. 2007; Yu et al. 2015; Liu et al. 2017; Richter et al. 2019; Hemmerling, Ackermann, and Ruess 2023) and thus modulate the composition of bacterial soil communities by their grazing activity (Xiao et al. 2014; Jiang et al. 2017; Brondani et al. 2022). These pioneering insights were restricted to bacteria easy to grow in the laboratory. However, major bacterial (e.g., *Acidobacteriota*, *Gemmatimonadota*) and archaeal (*Nitrososphaerota*) taxa dominating in soil (Leininger et al. 2006; Delgado‐Baquerizo et al. 2018) were so far neglected.

Our experimental setup allowed us to decipher population dynamics of dominant bacterial and archaeal species‐level ASVs exposed to grazing by a typical bacterivorous soil nematode, *A. buetschlii* , and how this feeds back on soil respiration dynamics. Addition of *A. buetschlii* clearly increased soil respiration at the beginning of the experiment and shifted its maximum rate from approximately day 12 to day 6 if compared to treatments without *A. buetschlii* addition. Although we cannot exclude a synergistic effect of the slightly higher water content in treatments with *A. butschlii* addition, our data are in line with previous studies that showed alteration of soil respiration by bacterivorous nematodes (Xiao et al. 2014; Zhu et al. 2018; Richter et al. 2019). Therefore, we interpret the shifts in soil respiration dynamics as a well distinguishable top‐down control signal. Irrespective of *A. buetschlii* addition, maize litter significantly (*p* < 0.05) increased soil respiration rates corresponding to a well distinguishable bottom‐up control signal. The combination of *A. buetschlii* population development (Figure 1A), soil respiration dynamics in the presence of *A. buetschlii* addition (Figure 1B), the time‐resolved magnitude of bacterial and archaeal community changes (Figure 2A and Figure S4) and the population response type analysis of bacterial and archaeal ASVs (Figure 3) showed that the first 8 days of the experiment were decisive to understand predator–prey effects. Importantly, within these first 8 days control incubations without *A. buetschlii* addition had throughout negligible nematode populations of all trophic groups including bacterivorous nematodes (Figure 1A), had lower soil respiration rates (Figure 1B) and showed less pronounced bacterial and archaeal community changes (Figure 2A and Figure S4). Taken together, this clearly showed a measurable top down effect by *A. buetschlii* addition and bottom up effect of maize addition on the bacterial and archaeal community. Since *A. buetschlii* populations steadily increased from day 8 onwards, top‐down effects can be safely attributed to grazing activity.

Comparison of the isolated effect of added bacterivorous *A. buetschlii* (+A/−M treatment) to treatments without *A. buetschlii* addition (−A/+M; −A/−M) allowed us to identify bacterial and archaeal species‐level taxa that clearly declined in population size (response types A and B) in the presence of a dominant *A. buetschlii* population but benefited from extremely reduced bacterivorous nematode populations including *A. buetschlii* (response types C and D), irrespective of maize litter addition. It was evident that some of the most abundant bacterial ASVs in the analysed soil were negatively affected by *A. buetschlii* . Specifically, they were represented in large parts by the four most prominent response type patterns observed in terms of summed median abundance (Figure 4). To a minor extent, dominant ASVs grouped in less prominent response type patterns contributed as well (Figure S5, Table S5). Although we cannot differentiate between direct and indirect effects of grazing, *A. buetschlii* was likely feeding on major populations of very abundant Gram‐negative bacteria including the very prominent groups of *Acidobacteriota*, *Bacteriodota*, *Gemmatimonadota* and *Pseudomonadota*. At the same time, *A. buetschlii* addition did not affect abundant populations of Gram‐positive *Actinobacteriota* and *Bacillota* and some Gram‐negative bacteria related ASVs, e.g., within the *Comamonadaceae* and *Xanthomonadaceae* (Figure 4). This strongly indicates that the bacterivorous *A. buetschlii* does display feeding preferences also in its natural environment as already indicated by targeted experiments with model bacteria (Salinas et al. 2007; Yu et al. 2015; Liu et al. 2017).

The very abundant group of ammonia‐oxidising archaea (*Nitrososphaeraceae* ASVs) and their syntrophic partners, the nitrite‐oxidising bacteria (*Nitrospira* ASVs), were negatively affected in population size as well when comparing the isolated effect of *A. buetschlii* addition (+A/−M treatment) to treatments without *A. buetschlii* addition (−A/+M; −A/−M). Bacterivorous nematodes are well known to enhance soil N mineralization rates and thus provide excess ammonia as the primary substrate for the nitrifiers mentioned above (Anderson et al. 1983; Ingham et al. 1985; Ferris et al. 1998; Gebremikael, Buchan, and De Neve 2014; Holajjer, Kamra, and Singh 2016). As a consequence, one should expect population increases of these nitrifiers or neutral effects (Bonkowski, Villenave, and Griffiths 2009). The fact that these prominent nitrifier groups declined in abundance over the first eight days and only recovered afterwards (response type B) in the +A/−M treatment indicates that they served as primary food source of *A. buetschlii* as well. This palatability towards filter‐feeding would agree well with the generally small coccoid cell size of species belonging to the abundant archaeal *Nitrososphaeraceae* (≤ 1.1 μm, Stieglmeier et al. 2014; Lehtovirta‐Morley et al. 2016) and the slightly larger and spiral‐shaped bacterial *Nitrospira* (0.2–0.4 × 0.9–2.2 μm, Spieck and Bock 2015).

### 4.2 Top‐Down Control Induced Succession of Bacteria and Archaea Under Substrate Availability

**FIGURE 5:**
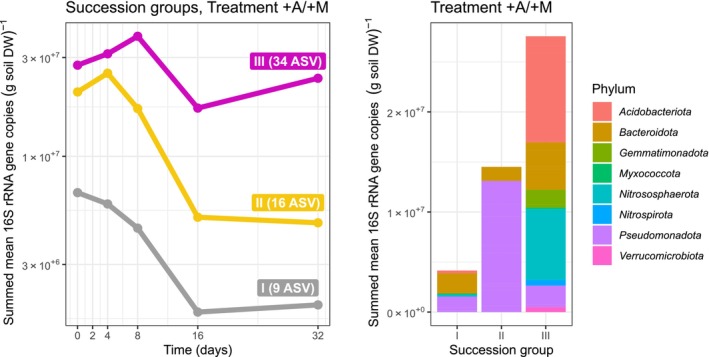
Succession of dominant bacterial and archaeal response ASVs under top‐down control by *A. buetschlii* grazing and simultaneous bottom‐up control by maize litter addition. Succession groups represent ASVs that were susceptible to *A. buetschlii* grazing (abundance decline within the first 8 days in the +A/−M but not in the −A/+M and −A/−M treatments) but split into response types A (succession group I), response type D (succession group II) and response type E (succession group III) in the +A/+M treatment according to Figure 3. Colour codes correspond to Figure 3. (A) Temporal dynamics of succession groups. (B) Affiliation and mean 16S rRNA gene copy numbers of corresponding ASVs across all time points of the +A/+M treatment. The mean was used to indicate the baseline abundance across all time points.

The simultaneous treatment with the bacterivorous nematode *A. buetschlii* and maize litter (+A/+M treatment) added another layer of complexity to our experimental system. It now involved both a top‐down and a bottom‐up control at the same time, which is a scenario close to natural settings in agricultural soils after harvest. ASVs that were susceptible to *A. buetschlii* grazing alone (as described in the discussion above) split now into three successional groups:

1. ASVs in succession group I declined in abundance in both *A. buetschlii* treatments (response type A), with and without added maize litter, and thus did not benefit from the applied bottom‐up control. It was composed of 9 ASVs, which constituted in sum 4.5% of the overall bacterial and archaeal community at the onset of the experiment (Figure 5A). Within the +A/+M treatment, they were mainly dominated by representatives of the *Bacteroidota* as deduced from their mean absolute abundance across all time points (Figure 5B).
2. ASVs in succession group II shifted from population declines under the +A/−M treatment (response types A and B) to an intermediate abundance peak at day 4 (response type D) in the +A/+M treatment. This indicates that these ASVs benefited from substrate addition (bottom‐up control) despite simultaneous grazing (top‐down control) until day 4, which marked the turning point of a stronger top‐down control. Succession group II was composed of 16 ASVs, which constituted 13.8% of the overall bacterial and archaeal community at the onset of the experiment (Figure 5A). They were heavily dominated by *Pseudomonadota* in terms of mean absolute abundance throughout the +A/+M treatment (Figure 5B).
3. Interestingly, there was a third succession group, where the turning point from stronger bottom‐up to stronger top‐down control was extended to day 8. Succession group III was composed of even more ASVs (34 in total), which constituted 18.6% to the overall bacterial and archaeal community at the onset of the experiment (Figure 5A). These 34 ASVs were spanning seven different phyla with representatives of the *Acidobacteriota* (domain *Bacteria*) and *Nitrososphaerota* (domain *Archaea*) dominating in terms of mean absolute abundance throughout the +A/+M treatment (Figure 5B). In summary, this revealed that *A. buetschlii* grazing under simultaneous substrate availability induces a succession of major bacterial and archaeal soil taxa within the soil micro food‐web.

## 5 Conclusion

Soil respiration constitutes a major carbon flux between the land surface and the atmosphere. Grazing by the bacterivorous *A. buetschlii* clearly accelerated soil respiration and, consequently, the underlying carbon flow within the soil. This effect was centred on a broad feeding behaviour targeting dominant populations of Gram‐negative bacteria and ammonia‐oxidising archaea, while discriminating against dominant populations of Gram‐positive bacteria. Under simultaneous bottom‐up control by maize litter addition, top‐down control by nematode grazing caused a succession of microbial groups. This succession was likely driven by a combination of different microbial resource use efficiencies, specialisation for substrates with varying degradability and grazing‐induced release of degradation products such as ammonia. This emphasises the importance of the micro‐food web for nitrogen‐use efficiency in soils, impacting the conversion of ammonia to nitrate by nitrifier activity. Our temporally resolved succession model provides a first understanding of nematode grazing down to the bacterial and archaeal species level. It lays conceptual grounds for model approaches that result in predictive ecology of soils, benefiting sustainable agriculture.

## Author Contributions

**Mandip Tamang:** writing – original draft, investigation, formal analysis. **Johannes Sikorski:** writing – review and editing, formal analysis, data curation. **Miriam van Bommel:** investigation, writing – review and editing. **Marc Piecha:** investigation, writing – review and editing. **Tim Urich:** conceptualization, writing – review and editing, supervision, funding acquisition. **Liliane Ruess:** conceptualization, writing – review and editing, supervision, funding acquisition. **Katharina Huber:** conceptualization, writing – review and editing, supervision, funding acquisition. **Meina Neumann‐Schaal:** conceptualization, writing – review and editing, supervision, funding acquisition. **Michael Pester:** conceptualization, funding acquisition, writing – original draft, writing – review and editing, supervision, project administration.

## Conflicts of Interest

The authors declare no conflicts of interest.

## Supporting information


Data S1.

## Data Availability

All amplicon sequences were deposited at the Sequence Read Archive at the National Center for Biotechnology Information (NCBI) under the Bioproject number PRJNA1092885. Table S5 was deposited at Zenodo under the following doi: https://doi.org/10.5281/zenodo.14044359.
