# Supplementary material for: Succession of Bacteria and Archaea Within the Soil Micro‐Food Web Shifts Soil Respiration Dynamics
Source: Environ Microbiol. 2024 Nov 21;26(11):e70007. doi: 10.1111/1462-2920.70007 (PMC11582019; doi:10.1111/1462-2920.70007)
Supplement: Supplementary file 1 — Data S1. [file EMI-26-e70007-s001.pdf]

Appendix

Succession of bacteria and archaea within the soil micro-food web shifts soil respiration dynamics

Mandip Tamang[1], Johannes Sikorski[1], Miriam van Bommel[2], Marc Piecha[3], Tim Urich[3], Liliane Ruess[2], Katharina Huber[1], Meina Neumann-Schaal[1, 4] and Michael Pester[1, 4, 5*]

1. *Leibniz Institute DSMZ – German Collection of Microorganisms and Cell Cultures, Inhoffenstr. 7B, D-38124 Braunschweig, Germany*
2. *Humboldt-Universität zu Berlin, Institute of Biology, Ecology Group, Philippstraße 13, 10115, Berlin, Germany*
3. *University of Greifswald, Institute of Microbiology, Felix-Hausdorff-Str. 8, D-17487 Greifswald, Germany*
4. *Braunschweig Integrated Centre of Systems Biology (BRICS), Rebenring 56, D-38106 Braunschweig*
5. *Technical University of Braunschweig, Institute for Microbiology, Spielmannstrasse 7, D-38106 Braunschweig, Germany*

*Corresponding author: Michael Pester, Mail: Michael.Pester@dsmz.de, Phone: +49 531 2616237

Supplementary Tables

Supplementary Table 1. Results of model selection for possible generalized additive models (gam) relating soil respiration to incubation time (day), bacterivorous *A. buetschlii* addition (treatment), and maize litter addition (substrate).

Model	Modeled response under	Deviance expplained (%)	deltaAIC
mod0 <- gam(respiration ~ day)	no smooth. Null model.	4.11	783.9
mod_G <- gam(respiration ~ s(day))	global smooth for day.	32.2	672.0
mod_GI <- gam(respiration ~ s(day) + s(day, by=treatment, bs=”tp”)	global smooth for day *plus* individual group smooths per day for nematode treatment.	87.2	72.6
mod_I <- gam(respiration ~ s(day, by=treatment, bs=”tp”)	individual group smooths per day for nematode treatment.	87.2	72.6
mod_GS <- gam(respiration ~ s(day) + s(day, by=treatment, bs=”fs”)	global smooth for day *plus* shared group smooths per day for nematode treatment.	87.2	72.6
mod_S <- gam(respiration ~ s(day, by=treatment, bs=”tp”)	shared group smooths per day for nematode treatment.	87.2	72.6
modS_ST <- gam(respiration ~ substrate + treatment + s(day, by=treatment, bs=”fs”)	additive effects of substrate addition, treatment type, and shared group smooths per day for nematode treatment.	89.5	1.9
modS_SintactT <- gam(respiration ~ substrate*treatment + s(day, by=treatment, bs=”fs”)	interacting effects of substrate addition and treatment type plus shared group smooths per day for nematode treatment.	89.6	0.0

Diagnostic plots of the best fitting GAM (modS_SintactT) are given in Supplementary Figure 2.

Supplementary Table 2. Summary of the best-fitting GAM obtained used to explain soil respiration. The structure of the final model according to Supplementary Table 1 was modS_SintactT <- gam(respiration ~ substrate*treatment + s(day, by=treatment, bs=”fs”); method = REML, family = Gaussian.

Parametric coefficients

	Estimate	Std. error	t-value	*p*-value	Significance
Intercept	1.36421	0.03331	40.951	$< 2 \times 10^{-16}$	***
Substrate addition	0.11751	0.02338	5.025	8.05×10^{-07}	***
A. buetschlii treatment	0.06989	0.04362	1.602	0.1100	
Substrate : *A. buetschlii*	0.06661	0.03413	1.952	0.0518	.

Significance codes: 0 ‘***’ ; 0.001 ‘**’ ; 0.01 ‘*’ ; 0.05 ‘.’ ; 0.1 ‘ ‘ ; 1

Approximate significance of smooth terms

	edf	Ref.df	F	*p*-value	Significance
s(day,treatment)	15.74	16	170.2	$< 2 \times 10^{-16}$	***

Significance codes: 0 ‘***’ ; 0.001 ‘**’ ; 0.01 ‘*’ ; 0.05 ‘.’ ; 0.1 ‘ ‘ ; 1

R^2 (adj) = 0.89, deviance explained = 89.6%

-REML = -90.74, scale est. = 0.026648, n = 369

Supplementary Table 3. Analysis of variance (ANOVA) of total abundance, alpha diversity (^{0}D and ^{2}D), and rank abundance structure (alpha-gambin value) changes over time. A – *A. buetschlii*; M – maize litter. Significant results at $p < 0.05$ are given in bold.

Parameter	Treatment	$F_{4,10}$-value	*p*-value
Total bacterial and archaeal 16S rRNA genes/g dry soil	**+A/+M**	**4.780**	**0.048**
	+A/–M	0.316	0.583
	–A/+M	2.029	0.178
	–A/–M	0.001	0.907
ASV richness (^{0}D)	+A/+M	0.815	0.383
	+A/–M	1.370	0.262
	–A/+M	0.662	0.431
	–A/–M	0.632	0.441
Dominant ASVs (^{2}D)	+A/+M	0.403	0.537
	+A/–M	1.290	0.277
	–A/+M	0.127	0.727
	–A/–M	1.530	0.238
Alpha-gambin	+A/+M	0.042	0.840
	**+A/–M**	**8.450**	**0.012**
	–A/+M	0.015	0.903
	**–A/–M**	**6.370**	**0.025**

Supplementary Table 4. Post-hoc comparisons of group means for significant ANOVA results shown in Supplementary Table 3. Post-hoc tests were done using the R package multcomp.

Total bacterial and archaeal 16S rRNA genes (g dry soil)$^{-1}$, Treatment +A/+M

Linear Hypotheses	Estimate	Std. Error	t value	Adjusted p-value
4 - 0 == 0	3.61E+07	2.27E+07	1.590	0.501
8 - 0 == 0	5.88E+07	5.18E+07	1.136	0.760
16 - 0 == 0	-5.42E+07	1.95E+07	-2.780	0.096
32 - 0 == 0	-3.82E+07	2.15E+07	-1.773	0.405
8 - 4 == 0	2.27E+07	5.00E+07	0.453	0.988
**16 - 4 == 0**	**-9.03E+07**	**1.42E+07**	**-6.351**	**0.001**
**32 - 4 == 0**	**-7.43E+07**	**1.69E+07**	**-4.391**	**0.008**
16 - 8 == 0	-1.13E+08	4.86E+07	-2.323	0.192
32 - 8 == 0	-9.70E+07	4.95E+07	-1.960	0.319
32 - 16 == 0	1.60E+07	1.23E+07	1.305	0.665

Alpha-gambin, Treatment +A/-M

Linear Hypotheses	Estimate	Std. Error	t value	Adjusted p-value
4 - 0 == 0	2.88E-03	0.200	0.014	1
8 - 0 == 0	-1.43E-01	0.184	-0.775	0.924
16 - 0 == 0	-1.76E-01	0.182	-0.965	0.851
32 - 0 == 0	-2.67E-01	0.175	-1.530	0.542
8 - 4 == 0	-1.46E-01	0.122	-1.199	0.732
16 - 4 == 0	-1.79E-01	0.119	-1.509	0.554
32 - 4 == 0	-2.70E-01	0.106	-2.541	0.143
16 - 8 == 0	-3.32E-02	0.090	-0.369	0.995
32 - 8 == 0	-1.25E-01	0.073	-1.702	0.448
32 - 16 == 0	-9.14E-02	0.068	-1.341	0.651

Alpha-gambin, Treatment -A/-M

Linear Hypotheses	Estimate	Std. Error	t value	Adjusted p-value
4 - 0 == 0	-2.07E-01	0.196	-1.058	0.811
8 - 0 == 0	-4.26E-01	0.248	-1.718	0.446
16 - 0 == 0	-2.60E-01	0.334	-0.780	0.925
32 - 0 == 0	-5.75E-01	0.208	-2.770	0.104
8 - 4 == 0	-2.19E-01	0.187	-1.172	0.751
16 - 4 == 0	-5.30E-02	0.291	-0.182	1
32 - 4 == 0	-3.68E-01	0.128	-2.869	0.089
16 - 8 == 0	1.66E-01	0.328	0.505	0.983
32 - 8 == 0	-1.49E-01	0.199	-0.749	0.934
32 - 16 == 0	-3.15E-01	0.299	-1.053	0.813

Supplementary Table 5. Overview over abundance changes, taxonomic affiliation, and response type of dominant ASVs under the different treatment combinations. M – maize litter, A – *A. buetschlii*, + and – signs indicate presence and absence, respectively. The file can be accessed via Zenodo under the following doi: https://doi.org/10.5281/zenodo.14044359.

Supplementary Figures

Supplementary Figure 1. Overview of experimental design. The chart was created with BioRender.com under license number QQ276VNTCH for use in journal publications.

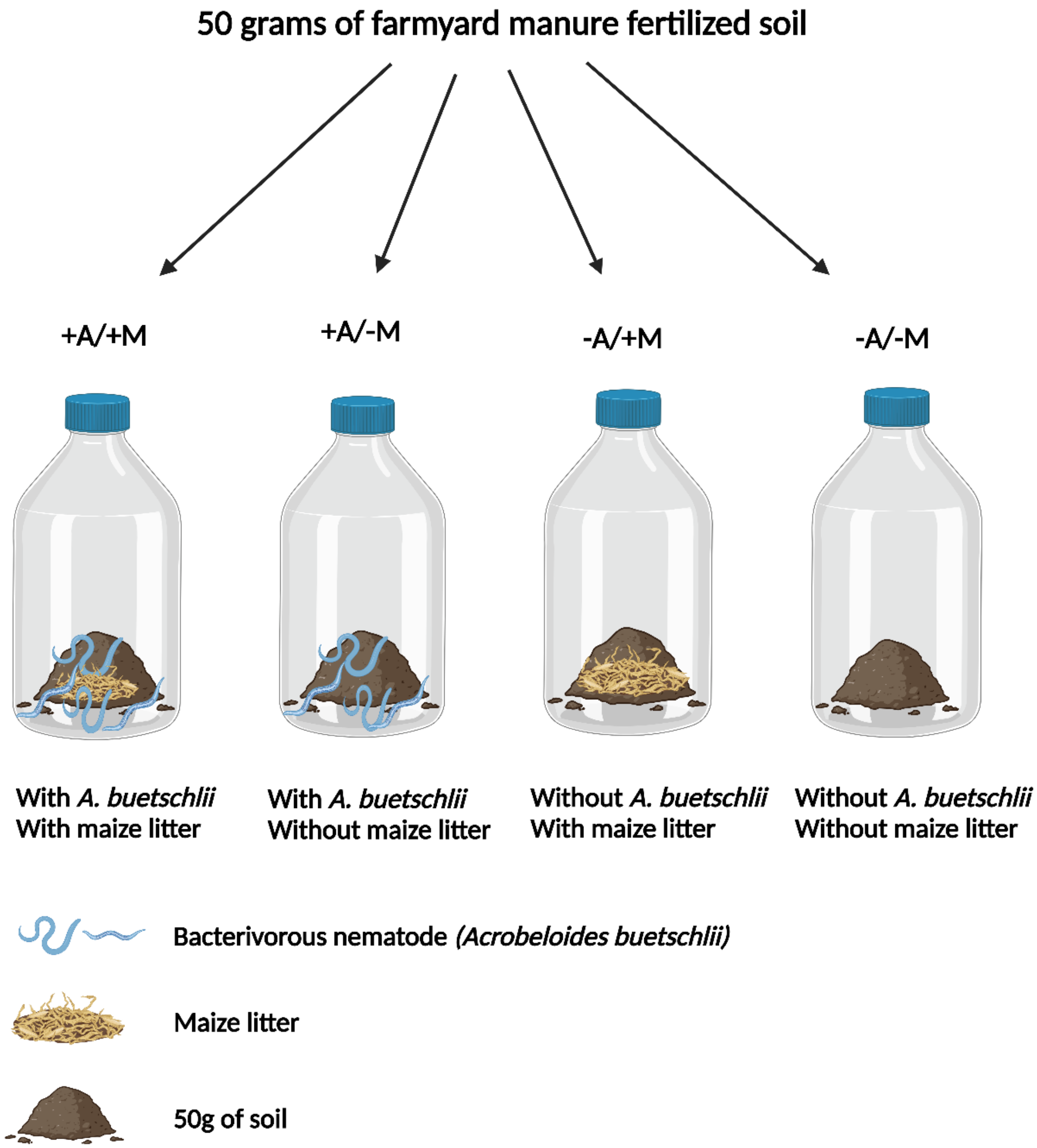
50 grams of farmyard manure fertilized soil
+A/+M
+A/-M
-A/+M
-A/-M
With *A. buetschlii*
With maize litter
With *A. buetschlii*
Without maize litter
Without *A. buetschlii*
With maize litter
Without *A. buetschlii*
Without maize litter
Bacterivorous nematode *(Acrobeloides buetschlii)*
Maize litter
50g of soil

Supplementary Figure 2. Diagnostic plots of the best fitting GAM (modS_SintactT) used to explain soil respiration in relation to day, treatment, and substrate

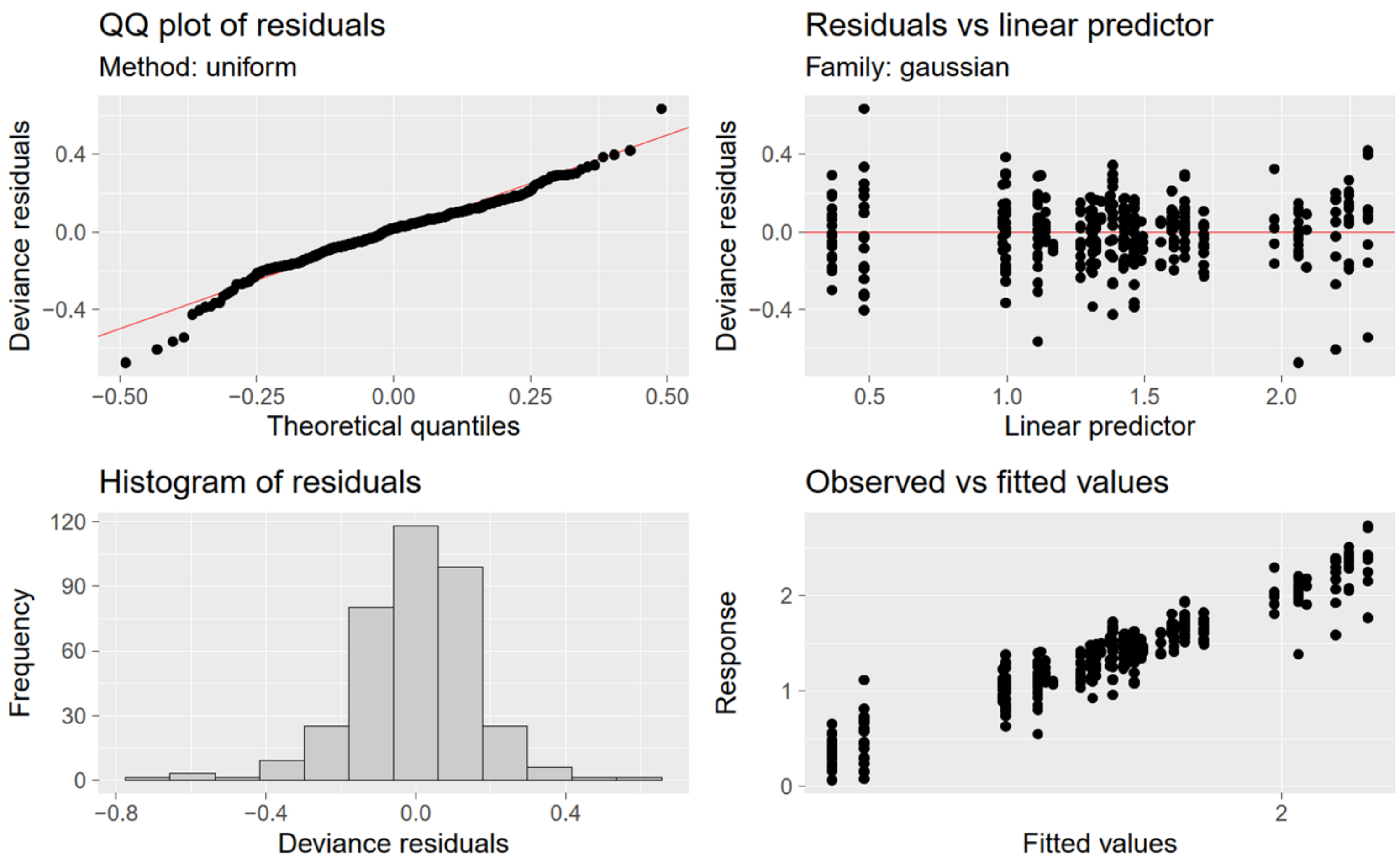

Supplementary Figure 3. Absolute abundance and alpha diversity of total Bacteria and Archaea throughout the experiment. Alpha diversity was based on Hill number analysis (Alberdi and Gilbert, 2019a; Alberdi and Gilbert, 2019b). The alpha-gambin value is provided as a parameter describing rank abundance distributions (Matthews et al., 2014).

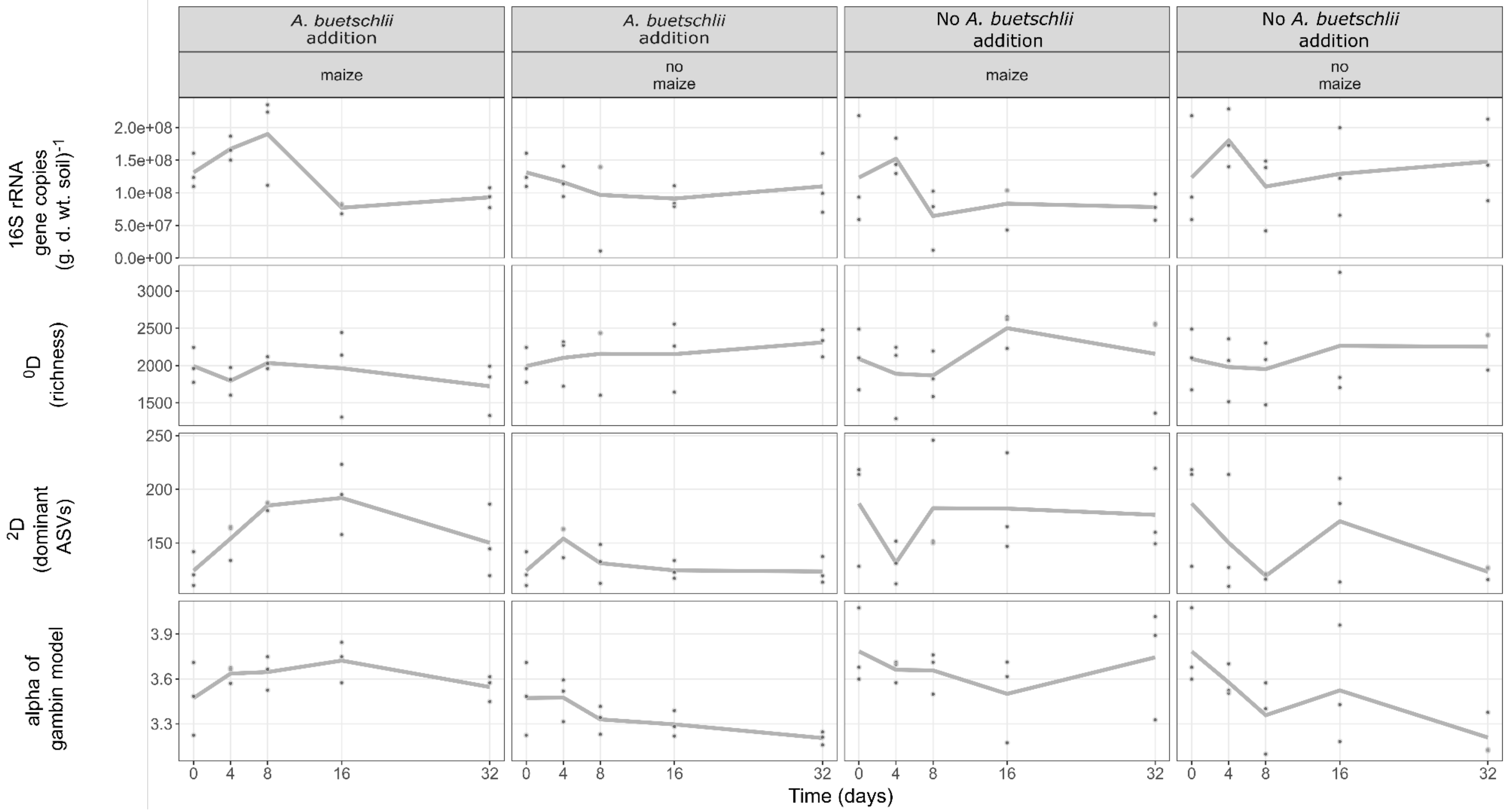

Supplementary Figure 4. Changes of the Aitchison beta diversity metrics over time in response to addition of the bacterivorous nematode *A. buetschlii* and maize litter. Beta-diversity distances were calculated between all pairs of replicates. Each boxplot represents nine pairwise comparisons (three replicates of one time point compared to all three replicates of another time point). Only the boxplot at time point 0 is covered by 15 data points (three pairs of replicate comparison per time point, five time points).

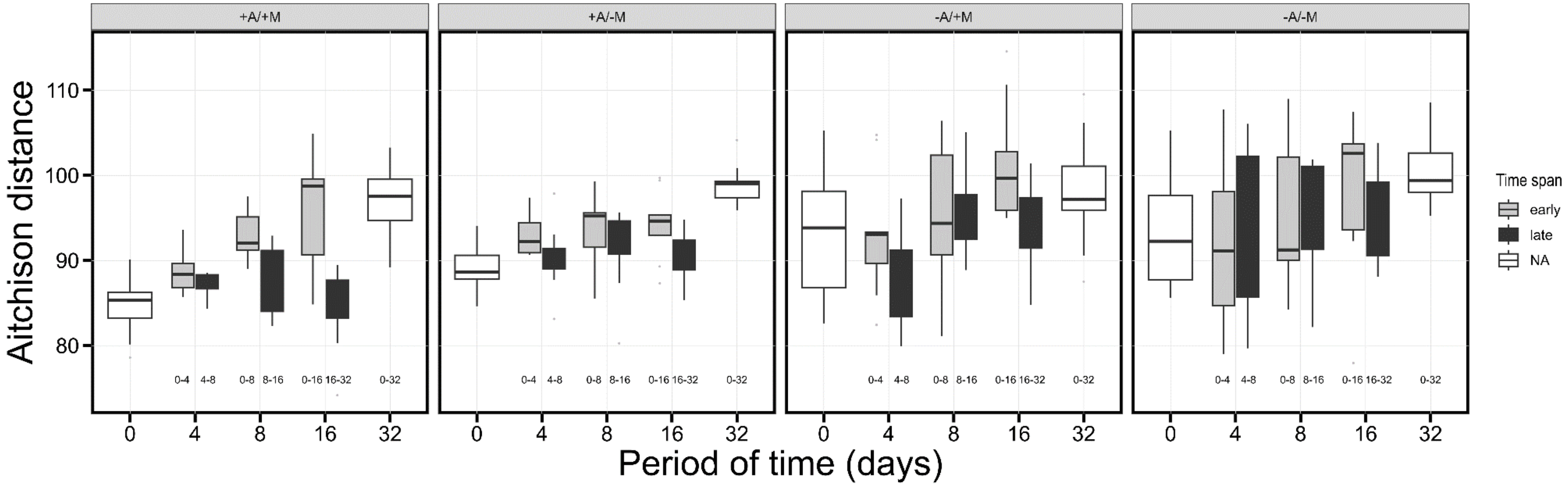

Supplementary Figure 5. Rank abundance distribution of all observed response type patterns as based on the summed median abundances of their representing ASVs. Response types of individual ASVs are shown in Supplementary Table 5. Letters correspond to response types shown in Fig. 3. If representing ASVs did not belong to a certain response type under a given treatment "_" is indicated. The sequence of letters corresponds to treatments in the following order: +A/+M, +A/−M, −A/+M, −A/−M.

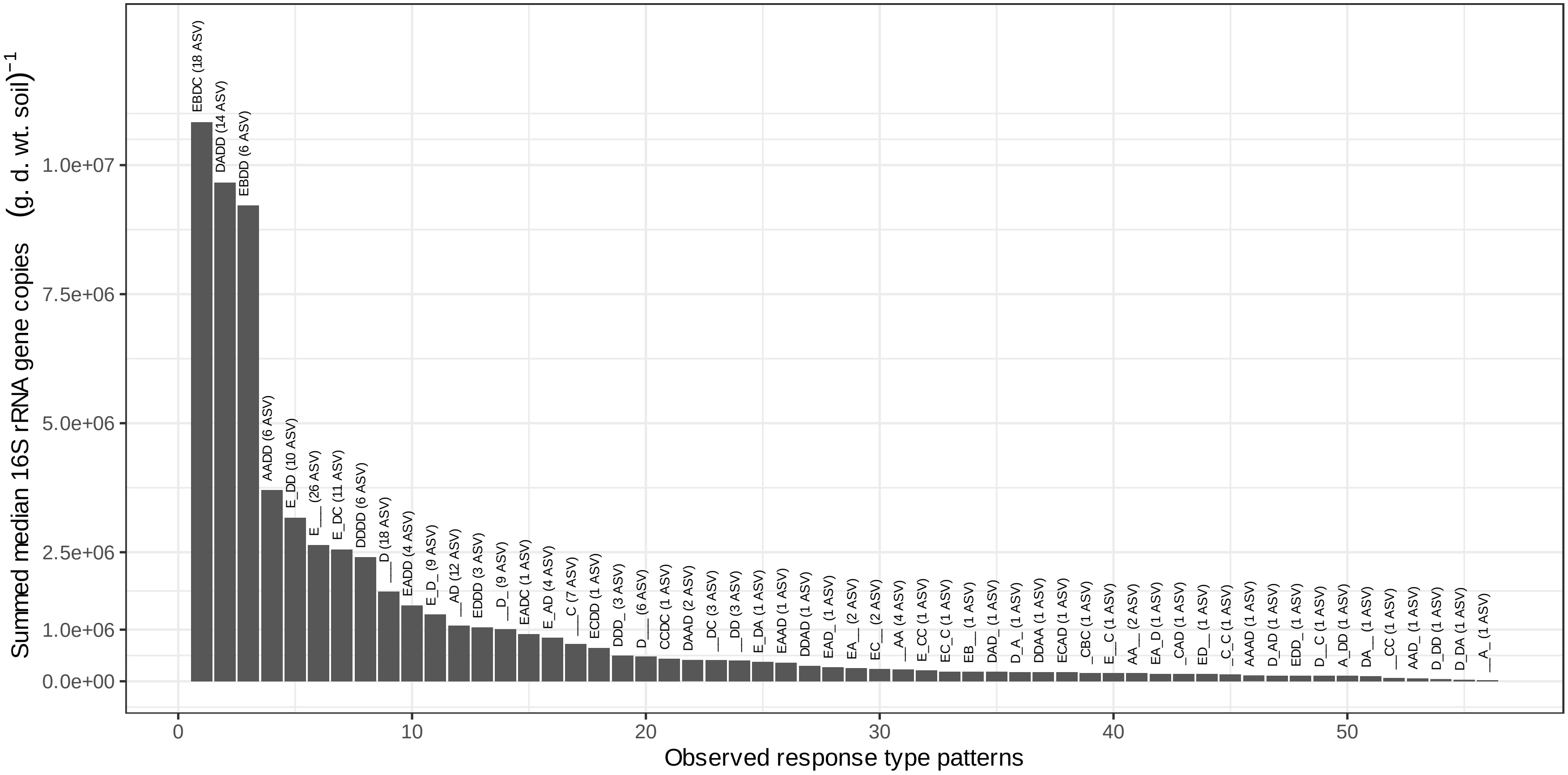

References

Alberdi, A., and Gilbert, M.T.P. (2019a) hilldiv: an R package for the integral analysis of diversity based on Hill numbers. *bioRxiv*: 545665.

Alberdi, A., and Gilbert, M.T.P. (2019b) A guide to the application of Hill numbers to DNA-based diversity analyses. *Molecular Ecology Resources* **19**: 804-817.

Matthews, T.J., Borregaard, M.K., Ugland, K.I., Borges, P.A.V., Rigal, F., Cardoso, P., and Whittaker, R.J. (2014) The gambin model provides a superior fit to species abundance distributions with a single free parameter: evidence, implementation and interpretation. *Ecography* **37**: 1002-1011.
